# Acute Pancreatitis Unmasking an Ectopic Parathyroid Adenoma in MEN1 Syndrome and Cushing’s Syndrome: A Rare Clinical Convergence

**DOI:** 10.15388/Amed.2026.33.1.22

**Published:** 2026-07-22

**Authors:** Asif Dabeer Jafri, Om Prakash Sanjeev, Aftab Hasan Nazar, Ratender Kumar Singh

**Affiliations:** Department of Emergency Medicine, Sanjay Gandhi Post Graduate Institute of Medical Sciences, Lucknow, Uttar Pradesh, India

**Keywords:** Multiple Endocrine Neoplasia Type 1 (MEN1), Ectopic Parathyroid Adenoma, Hypercalcemia, Acute Pancreatitis, dauginė endokrininė neoplazija, 1 tipo (MEN1), ektopinė prieskydinių liaukų adenoma, hiperkalcemija, ūminis pankreatitas

## Abstract

Ectopic parathyroid adenoma within the spectrum of Multiple Endocrine Neoplasia Type 1 (MEN1) is an extremely rare clinical entity. We report a 27-year-old woman who presented with *acute pancreatitis* as the sentinel manifestation of MEN1, an atypical and intriguing clinical scenario. Initially, her serum calcium levels were deceptively normal, only to surge later, unmasking a deeper endocrine pathology. Subsequent biochemical evaluation established *primary hyperparathyroidism (PHPT)*, and a methoxyisobutylisonitrile (MIBI) scan revealed an ectopic parathyroid adenoma as the culprit lesion. Further endocrine profiling disclosed *Cushing’s syndrome (CS)*, confirmed through a low-dose dexamethasone suppression test, while a positive family history of MEN1-related tumors in her sister solidified the syndromic diagnosis. She was treated with aggressive hydration, analgesia, antibiotics, and supportive therapy, followed by successful parathyroidectomy.

This case illustrates a remarkable convergence of conditions including ectopic parathyroid adenoma, acute pancreatitis, and MEN1 with the additional complexity of initially normocalcemic presentation during the acute phase of pancreatitis. Although the pathophysiological link between hypercalcemia and pancreatitis remains debated, pancreatitis secondary to hypercalcemia from primary hyperparathyroidism is rare, and its presentation as the first clue to MEN1 is exceptionally uncommon. ACTH-secreting pituitary adenomas causing CS are also a rare manifestation of MEN1, and the concomitant presence of an empty sella on brain Magnetic resonance imaging (MRI) further underscores the uniqueness of this case. To the best of our knowledge, this represents the first documented instance in which acute pancreatitis with normocalcemia concealed an underlying ectopic parathyroid adenoma in MEN1, accompanied by CS and an empty sella, making this a noteworthy clinical and diagnostic rarity.

## Abstract (it)

Ektopinė prieskydinių liaukų adenoma, pasireiškianti kaip 1 tipo dauginės endokrininės neoplazijos (MEN1) simptomas, yra itin retas klinikinis atvejis. 27 metų moteriai ūminis pankreatitas pasireiškė kaip pirminis MEN1 simptomas – tai netipiškas ir įdomus klinikinis atvejis. Iš pradžių pacientės kalcio koncentracija serume buvo apgaulingai normali, tačiau vėliau staigiai pakilo, tai atskleidė gilesnę endokrininę patologiją. Vėlesniu biocheminiu tyrimu patvirtintas pirminis hiperparatiroidizmas (PHPT), o MIBG tyrimas parodė, kad yra ir ektopinė prieskydinių liaukų adenoma. Tolesnis endokrinologinis tyrimas atskleidė esant Kušingo sindromą (CS), patvirtintą mažos dozės deksametazono slopinimo testu, o teigiami šeimos anamnezės duomenys apie su MEN1 susijusius navikus pacientės seseriai sustiprino sindromo diagnozę. Moteriai buvo taikoma intensyvi rehidratacija, analgezija, ji gydyta antibiotikais ir skirtas palaikomasis gydymas, paskui sėkmingai atlikta paratireoidektomija.

Šis atvejis iliustruoja neįtikėtiną ligų, įskaitant ektopinę prieskydinių liaukų adenomą, ūminį pankreatitą ir MEN1, sutaptį, kurią dar labiau apsunkina pradinis normokalceminis vaizdas ūminio pankreatito fazėje. Nors patofiziologinis hiperkalcemijos ir pankreatito ryšys tebėra ginčytinas, pankreatitas, antrinis dėl pirminio hiperparatireoidizmo sukeltai hiperkalcemijai, yra retas, o jo reiškimasis kaip pirmoji užuomina apie MEN1 yra ypač retas. ACTH išskiriančios hipofizės adenomos, sukeliančios Kušingo sindromą, taip pat yra reta MEN1 raiška, o kartu tuščioji sella MRT dar labiau pabrėžia šio atvejo unikalumą. Kiek mums žinoma, tai yra pirmasis užfiksuotas atvejis, kai ūminis pankreatitas su normokalcemija paslėpė pagrindinę ektopinę paratireoidinę adenomą MEN1 atveju, kurią lydėjo Kušingo sindromas ir tuščios sellos sindromas, todėl tai yra reikšmingas ir retas klinikinis ir diagnostinis atvejis.

## Introduction

Multiple Endocrine Neoplasia Type 1 (MEN1), also known as Wermer syndrome, is caused by mutations in the MEN1 gene on chromosome 11q13, which encodes the tumor suppressor protein menin. It follows an autosomal dominant inheritance pattern and leads to the development of endocrine tumors, primarily affecting the parathyroid, pituitary, and pancreatic glands [1]. The estimated prevalence of MEN1 is approximately 1 in 30,000 individuals, with a highly variable clinical presentation depending on the specific endocrine organs involved [2]. Primary hyperparathyroidism (PHPT) represents the most common, and often the earliest manifestation, occurring in nearly 90% of MEN1 cases, typically due to multiglandular hyperplasia or adenomas [3]. However, ectopic parathyroid adenomas in MEN1 are exceptionally rare, posing significant diagnostic and clinical challenges [4,5].

Hypercalcemia is a defining characteristic of PHPT, which acts as a significant diagnostic indicator in MEN1. Nevertheless, certain conditions, such as acute pancreatitis, can complicate its detection. Although acknowledged, pancreatitis is a rare complication of hypercalcemia, occurring due to the activation of trypsinogen in pancreatic acinar cells triggered by elevated calcium levels [6]. This case report describes a unique presentation of MEN1, where acute pancreatitis initially masked hypercalcemia, delaying the diagnosis of primary hyperparathyroidism.

## Case presentation

A 27-year-old female with no known prior comorbidities presented to Emergency Department (ED) with a one-month history of severe, continuous epigastric pain radiating to the back, associated with occasional episodes of vomiting. As episodes of vomiting and severity of pain increased, she reported to our ED. On examination, the patient was conscious, oriented, and afebrile. Her vital signs at admission were as follows: pulse rate of 108 beats/minute, blood pressure of 117/71 mmHg, respiratory rate of 20 breaths/minute, and Peripheral oxygen saturation (SPO2) of 97% on room air. Her weight was 63.1 kg, height 151 cm, and body mass index (BMI) was 27.6 kg/m^2^. There was no evidence of thyroid swelling, collagenoma, lipoma, jaw mass, finger swelling. Yet, acanthosis nigricans and cushingoid facies was present.

There was no past medical history of fractures, jaw swelling, polyuria, psychiatric abnormalities, headaches, double vision, hypertension, or diabetes. The patient denied any history of weight gain, galactorrhea, amenorrhea, visual field deficits, recurrent episodes of sweating, tremors, excessive hunger, seizures, or migratory skin rash. Her menstrual history was notable for oligomenorrhea since menarche, with menstrual cycles occurring every 45–60 days. Family history was significant for nephrolithiasis in a paternal uncle, sudden death of her father in his third decade due to pancreatitis, and insulinoma in a paternal sister. Given the clinical presentation, the patient was referred to endocrinology specialists for further evaluation of hyperglycemia and coexistent hypercalcemia.

Table 1 presents her laboratory parameters. Contrast-enhanced computed tomography (CECT) of the abdomen revealed a bulky pancreas, cholelithiasis, medullary nephrocalcinosis, and a pancreatic neuroendocrine tumor measuring 6 × 5 mm. Partial symptomatic improvement was noted with conservative management with IV fluids, analgesics, and broad-spectrum antibiotics. Neck ultrasonography revealed normal findings. Bone mineral density (BMD) assessment indicated low bone mass. Magnetic resonance imaging of the sella turcica revealed a partially empty sella with a focal hypoenhancing lesion in the right anterior aspect of the pituitary gland, measuring approximately 8 × 5.9 mm, consistent with a pituitary microadenoma. The pituitary stalk was seen in the midline, with no suprasellar extension or significant cavernous sinus invasion (Knosp grade 1, Wilson-Hardy grade 2A). Genetic testing revealed a heterozygous mutation in the MEN1 gene (c.549G>A), which is consistent with the diagnosis of multiple endocrine neoplasia type 1.

**Table 1:** Laboratory parameters at the time of admission

Analyte	Patient result	Reference value
HB	11.1	13-17 g/dL
TLC	16.3	4-11 x 10^9^/L
TPC	325	150-400 x 10^9^/L
S. Na	133	135-145 mEq/L
S. K	3.8	3.5-5 mEq/L
S. Cl	97.3	96-115 mEq/L
SGOT	23	5-40 U/L
SGPT	14	5-40 U/L
T. Bilirubin	0.51	0.0- 2.0 mg/dL
Alkaline phosphatase	396	40-129 IU/L
T. Protein	5.4	6.0-8.3 g/dL
S. Albumin	2.6	3.3-5.2 g/dL
Urea	5.5	13-45 mg/dL
Creatinine	0.65	0.5-1.5mg/dL
Calcium	9.6 (initially) 11.76 (later)	8.6-10.3 mg/dL
TSH	3.62	0.45 to 5.0 mIU/L
HbA1c	6.7%	<5.7
Prolactin	222.3	102-496 mIU/L
CRP	15	0.00-1.00 mg/dL
S. Cortisol 8 am	719	165–635 nmol/L
S. Cortisol 11 pm	568	<138 nmol/L
ACTH	16.79	1.6-13.9 pmol/L
PTH (pre-excision)	17.83	1.6-6.9 pmol/L
PTH (post-excision) at 5 and 15min	2.24 1.83	1.6-6.9 pmol/L
Vitamin D	16.37	50-250 nmol/L
Amylase	120	22-80 U/L
Lipase	87	6 – 38 U/L
Triglyceride	276	<150 mg/dL

*Note*. The following abbreviations were used: Hb: Hemoglobin; TLC: Total Leukocyte Count; TPC: Total Platelet Count; S. Na: Serum Sodium; S. K: Serum Potassium; S. Cl: Serum Chloride; SGOT: Serum Glutamic-Oxaloacetic Transaminase; SGPT: Serum Glutamic-Pyruvic Transaminase; TSH: Thyroid-Stimulating Hormone; HbA1c-Glycated Hemoglobin; CRP: C-Reactive Protein; ACTH: adrenocorticotrophic hormone; TSH: thyroid-stimulating hormone; PTH: Parathyroid hormone.

Initially, the patient presented with normocalcemia; however, after a few days, her serum calcium levels began to rise, raising suspicion of an underlying endocrinopathy. The coexistence of normocalcemia and subsequent hypercalcemia in the setting of pancreatitis and nephrolithiasis, along with acanthosis nigricans, thin skin, abdominal striae, and hyperglycemia, suggested an endocrine disorder. Further evaluation for hypercalcemia revealed primary hyperparathyroidism as evidenced by high serum parathyroid hormone, with an ectopic parathyroid adenoma detected on an Technetium-99m Methoxyisobutylisonitrile (MIBI) scan, as shown in Figure 1. The presence of thin, fragile skin, acanthosis nigricans, hyperglycemia, and proximal muscle weakness raised a clinical suspicion of Cushing’s syndrome (CS). This was biochemically confirmed by inadequate cortisol suppression on the overnight 1-mg dexamethasone suppression test (ONDST) and the 48-hour low-dose dexamethasone suppression test (LDDST), in conjunction with elevated plasma Adrenocorticotropic hormone (ACTH) dependent CS levels, which was consistent with ACTH-dependent Cushing’s syndrome.

**Figure 1:**
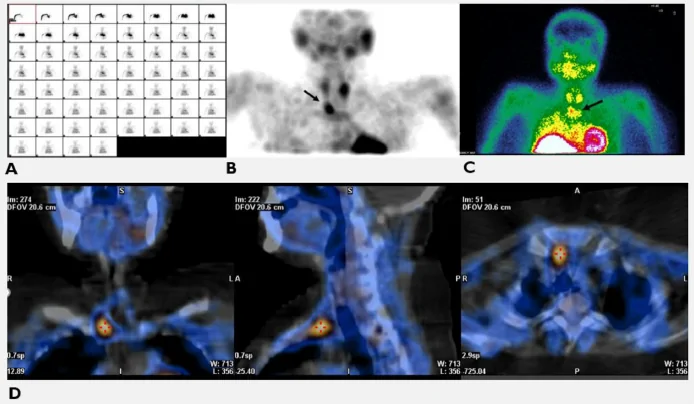
Perfusion images (A) demonstrate normal tracer flow in the neck. Early images (B) show physiological uptake in the thyroid gland with focal increased tracer uptake inferior to the right thyroid lobe (black arrow), which persists on delayed images despite thyroid washout. The Maximum intensity projection (MIP) image (C) localizes the lesion inferior to the right thyroid lobe (the black arrow). Single-photon emission computed tomography/computed tomography (SPECT/CT) images (D) of the neck and thorax reveal an ill-defined soft tissue lesion with focal tracer uptake posterior to the right sternoclavicular joint (red crosshair). These findings are suggestive of an ectopic parathyroid adenoma.

The patient was managed with intravenous fluids, analgesia (intravenous paracetamol 1 gram three times daily, intravenous tramadol 50 mg twice daily and as needed), intravenous ceftriaxone 2 grams twice daily, intravenous pantoprazole 40 mg once daily, intravenous ondansetron 4 mg three times daily for vomiting, and subcutaneous insulin. She subsequently underwent a parathyroidectomy for the ectopic parathyroid adenoma, followed by an endoscopic endonasal transsphenoidal resection of the pituitary microadenoma under general anesthesia. The procedure achieved gross total excision of the tumor, and her postoperative course was uneventful. With the administered treatment, gradual clinical improvement was observed. Following surgery, PTH and calcium levels normalized. A repeat CECT of the abdomen showed resolving features of pancreatitis. The patient remained afebrile following 15 days of intravenous antibiotic therapy and reported complete resolution of abdominal pain. She is under regular follow-up with the endocrinology department. Her younger brother and her son have been screened for mutation and were negative.

## Discussion

The occurrence of an ectopic parathyroid adenoma in MEN1 is extremely rare and poses a diagnostic challenge, primarily when initial normocalcemia masks the underlying condition. Ectopic parathyroid adenomas, which develop outside the typical anatomical sites, are infrequently encountered, representing less than 5% of all parathyroid tumors [7]. These adenomas are often found in the thymus, pericardium, or mediastinum. Accurate diagnosis and preoperative localization require advanced imaging techniques, including Technetium-99m sestamibi (99mTc-MIBI) scintigraphy. [8].

The clinical diagnosis of MEN1 can be confirmed in an individual who meets one of the following criteria: the presence of at least two endocrine tumors among the parathyroid, anterior pituitary, and well-differentiated neuroendocrine tumors of the gastrointestinal and pancreatic (GEP) tract, or the presence of one of these endocrine tumors (parathyroid, anterior pituitary, or well-differentiated neuroendocrine tumors of the GEP tract) along with a first-degree relative diagnosed with MEN1. Among anterior pituitary tumors, prolactinomas are the most frequently observed, accounting for around 60% of cases. Growth hormone-secreting adenomas follow at approximately 20%, while other less common variants include thyroid-stimulating hormone (TSH)-secreting adenomas, ACTH-secreting adenomas, and nonfunctional tumors. Well-differentiated neuroendocrine tumors of the GEP tract can develop in the stomach, duodenum, pancreas, and intestines, presenting with diverse clinical symptoms. Gastrinomas are the most frequently encountered, followed by insulinomas, glucagonomas, and vasoactive intestinal peptide-secreting tumors (VIPomas).

Hypercalcemia often leads to complications such as nephrolithiasis, osteoporosis, and various metabolic disorders [9]. In this particular case, the patient initially exhibited normocalcemia despite the presence of an ectopic parathyroid adenoma. This unusual situation can be explained by the concurrent acute pancreatitis, which may cause a temporary sequestration of calcium into necrotic fat due to saponification and decreased albumin levels resulting from systemic inflammation. The phenomenon of hypocalcemia associated with acute pancreatitis is well-documented, arising from the increased binding of calcium to fatty acids in pancreatic necrosis and a cytokine-mediated reduction in calcium levels [10]. This transient state of normocalcemia may have hindered the timely identification of the underlying PHPT, complicating the diagnostic process.

CS is an uncommon manifestation of MEN1 and may arise from either ACTH-dependent or ACTH-independent causes. Pituitary adenomas occur in approximately 40% of adults with MEN1, of which only 5 to 10% are ACTH-secreting and result in Cushing’s disease. In addition, ectopic ACTH production from thymic carcinoid tumors has been occasionally reported in association with MEN1, further expanding its endocrine spectrum [11]. CS represents an uncommon manifestation of MEN1, and the coexistence of an empty sella on MRI adds further distinction to this rare clinical presentation.

Long-term management of MEN1 requires regular monitoring of calcium and endocrine function to detect early tumor development. Genetic counseling and family screening are critical for the proactive identification and management of at-risk individuals.

## Conclusion

This case highlights the diagnostic complexity of MEN1, in which acute pancreatitis with initially normal serum calcium concealed an underlying ectopic parathyroid adenoma, a rare manifestation of MEN1, delaying the diagnosis of primary hyperparathyroidism. The concurrent presence of ACTH-dependent Cushing’s syndrome and a partially empty sella further illustrates the diverse clinical manifestations of MEN1. Clinicians should maintain a high index of suspicion for an underlying endocrine disorder in patients with acute pancreatitis accompanied by atypical clinical features or evolving hypercalcemia. Comprehensive biochemical, genetic, and advanced imaging evaluation is essential for timely diagnosis and definitive management.
